# Response: Commentary: Pupil old/new effects reflect stimulus encoding and decoding in short-term memory

**DOI:** 10.3389/fpsyg.2017.00539

**Published:** 2017-04-07

**Authors:** Andreas Brocher, Tim Graf

**Affiliations:** Institute of German Language and Literature I, University of CologneCologne, Germany

**Keywords:** pupil response, recognition memory, familiarity, recollection, old/new effect

Research on recognition memory has a long tradition in ERPs (see Yonelinas, 2002; Vilberg and Rugg, 2008 for reviews). One assumption is that recognition memory involves two distinct processes: familiarity and recollection. Familiarity refers to the feeling that a presented stimulus has been studied before, while recollection involves somewhat conscious retrieval of information associated with the respective study episode. Recently, Kafkas and Montaldi (2011, 2012) suggested that familiarity and recollection could also be distinguished in the size of the pupil. The claim is that the pupil dilates more for recollected than familiar stimuli. The distinction between familiarity and recollection in pupil size, however, is entirely based on remember/know paradigms. In Kafkas and Montaldi (2012), participants judged whether a presented stimulus was “old” or “new” and, in case of an “old” response, whether they can retrieve specific information of the study episode (“recall” response) or not (“familiar” response), and, in case of a “familiar” response, they then estimated the degree of familiarity (“weak” or “moderate” or “strong”).

In Brocher and Graf (2016), we argued that using a remember/know procedure is not ideal to unambiguously establish that pupil size distinguishes familiarity and recollection because it is difficult to estimate what exactly drives larger pupils in “remember” than “familiar” responses. It is possible that the observed differences obtain because participants engage in different processes *after* they made a response. Kafkas and Montaldi (2012) note that participants are “carefully trained to discriminate between instances of familiarity and recollection” (p. 3082). This means that participants are presumably highly sensitive to that discrimination and aware of the fact that it is crucial to their task. This point is important as it raises the possibility that, after pressing “recall,” participants engage in effortful retrieval to be sure that their response was correct. This could lead to larger pupils in “recollection” than “familiarity” responses (cf. Granholm et al., 1996; Granholm and Steinhauer, 2004).

In Brocher and Graf (2016), we conducted five pupil old/new experiments with materials that differently affect familiarity and recollection processes in ERPs. In all experiments, we used a simple old/new judgment task. Considering the well-established—albeit not perfect—similarities between EEG and pupil size data (Kuipers and Thierry, 2011, 2013; Kamp and Donchin, 2014; Scharinger et al., 2015), we reasoned that it might be possible to replicate the reported stimulus-driven effects with pupil size. What we found, however, was more compatible with a task- than a familiarity/recollection-related explanation of pupil old/new effects. All materials elicited robust old/new effects, with no additional effects of specific properties within the materials. More importantly, we found that old/new effects were significantly affected by the specific task participants engaged in.

Kafkas and Montaldi (2017) heavily criticized our study, mentioning four shortcomings, which we address below. We would first like to thank the authors for commenting on our research and, thereby, inspiring a discussion that we believe is important to fully understand pupil old/new effects. We would next like to admit that our use of “recall” and “short-term memory,” as Kafkas and Montaldi correctly note, are not compatible with the more traditional uses of these terms in memory research. In the case of the term “recall,” however, we believe that no deep misunderstanding should have occurred, since at no point in the description of our study do we state or infer that our participants were asked to recollect any information associated with study (see e.g., the description of the task on p. 1826). We also agree with Kafkas and Montaldi that our use of “short-term memory” is somewhat misleading. We do not use this term to refer to a Millerian concept of short-term memory (Miller, 1956), but to distinguish “short-term memory representations,” involving experiment-dependent representations, from representations that involve lifelong learning (“long-term memory representation”). To prevent any confusion, we should have carefully defined the two terms.

As a third point, Kafkas and Montaldi (2017) mention that we misinterpreted their results reported in Kafkas and Montaldi (2015). The authors object that they “clearly reported that the sensitivity of the pupil to old/new responses was maintained with simple decisions, and was therefore independent of task complexity” (p. 2). However, when comparing Figure 3B from their Experiment 1 (p. 1311) with Figure 4B from their Experiment 2 (p. 1312), it is obvious that the data from Experiment 2 do in fact *not fully* replicate the data from Experiment 1. Considering that the illustrated differences in misses are statistically reliable (the Task Response × Time interaction was only significant in Experiment 2), it is quite puzzling that Kafkas and Montaldi insist that they fully replicated the data from a remember/know task with a simpler old/new task. In fact, much of the conclusions of their data in the General Discussion concerns the distinction between objective and subjective memory processes, a distinction that is almost exclusively based on their findings from Experiment 2. Now, if the “only difference between the two experiments is the number of the available responses at retrieval” (p. 1307), what else could have led to the reported differences? Are their own data not also compatible with a task-sensitive approach to pupil old/new effects?

There are also methodological and statistical points in Kafkas and Montaldi (2015) that make it difficult to directly compare their Experiments 1 and 2 and fully appreciate the robustness of the reported effects. First, including a “recollection” option in Experiment 1 is problematic. We understand that this option was included “to ensure that they [participants] did not confound other response categories” (p. 1308). However, the main goal of the study was to distinguish processes in familiarity and processes in novelty detection. In the case of familiarity detection, recollection fully converges with the overall goal of the participant: Judge whether you have studied the stimulus onscreen. In contrast, in the case of novelty detection, recollection operates in the exact opposite of a participant's overall goal: Judge whether you have not studied the stimulus onscreen. Second, using an ANOVA is particularly problematic for Experiment 1, where the data are unlikely to be normally distributed within and across response categories, including detection strength. Third, in Experiment 2 the authors performed a series of (≥10!) paired *t*-tests on the time course data without any correction. The statistical approach, then, unnecessarily inflates the risk of eliciting false positives.

Turning to the fourth point of criticism, Kafkas and Montaldi mention that our “overarching assumption is that if a cognitive variable differentially affects the ERP components of familiarity and recollection, then it should also differentially affect the pupil responses” (p. 2). This is incorrect, we make clear that “we aim at bridging pupil and ERP old/new effects by analyzing pupil size for stimulus materials that have been shown to involve familiarity, recollection, or both in ERPs” (p. 1824). Thus, we do not *assume* that EEGs and pupil size reflect similar or even the same underlying processes. We simply take the stimulus-driven comparison between ERPs and pupil size as a *valid approach* toward collecting evidence supporting a familiarity/recollection distinction without confounding response and task demand. And, considering the evidence in favor of specific similarities between ERPs and pupil size, we believe this approach is fully legitimate. We also do not take the failure to replicate results from ERP with pupil size to show that pupil size cannot, in principle, distinguish familiarity and recollection. However, an alternative and, arguably, more parsimonious explanation is available. This explanation was tested in Brocher and Graf (2016), and the data of our Experiments 4 and 5 together with specific shortcomings of remember/know procedures do “challenge the view that pupil old/new effects can be directly mapped onto familiarity and/or recollection processes” (p. 1829).

On a more general level, Kafkas and Montaldi state that using the FN400 (associated with familiarity) and LPC (associated with recollection) in ERPs as “strong proxies for different forms of memory is unjustified” (p. 2). However, we argue that there are sufficient data available that do justify a systematic comparison between the two measures. To make that point clear, there is good evidence that pupil size distinguishes between the retrieval of low- and high-frequency words (Kuchinke et al., 2007; Papesh and Goldinger, 2012; Schmidtke, 2014), a finding that has long been established for ERPs. Why then would it be unjustified to include variables like these in a recognition memory task and compare them to ERPs? Considering the well-documented link between EEG and pupil size, there is, prima facie, no reason to believe that frequency similarly affects ERPs and pupil size in one kind of task, but not in another. And, in fact, does the claim that remember/know procedures elicit differences in old/new effects not also have an exact correlate in the ERP literature?

In sum, while we truly appreciate the comments in Kafkas and Montaldi (2017), since they caution us to be very precise in our wording and underlying assumptions, they have little bearing on the actual data that we presented in Brocher and Graf (2016). We believe that, in bringing the two lines of research together, we first need to standardize the implemented materials and statistical analyses. In a second step, then, it might be promising to design an experiment that first requires participants to distinguish studied from unstudied items, and only in response to a later and randomly occurring prompt would participants also indicate whether they recollect specific information or not. Irrespective of how a bridging study might look like, we hope that the present response initiates or, at least, inspires future work on pupil old/new effects, because, as we claim, these effects are as of now quite poorly understood.

## Author contributions

AB and TG wrote the commentary. AB and TG approved the paper for publication.

### Conflict of interest statement

The authors declare that the research was conducted in the absence of any commercial or financial relationships that could be construed as a potential conflict of interest.
